# CXCR4 attenuates cardiomyocytes mitochondrial dysfunction to resist ischaemia-reperfusion injury

**DOI:** 10.1111/jcmm.12554

**Published:** 2015-03-30

**Authors:** Wen-Feng Cai, Kai Kang, Wei Huang, Jia-Liang Liang, Yu-Liang Feng, Guan-Sheng Liu, De-Hua Chang, Zhi-Li Wen, Christian Paul, Meifeng Xu, Ronald W Millard, Yigang Wang

**Affiliations:** a Department of Pathology & Lab Medicine, College of Medicine, University of CincinnatiCincinnati, OH, USA; b Department of Pharmacology & Cell Biophysics, College of Medicine, University of CincinnatiCincinnati, OH, USA

**Keywords:** CXCR4 overexpression, ischaemia/reperfusion injury, mitochondria, cardiomyocytes, STAT3

## Abstract

The chemokine (C-X-C motif) receptor 4 (CXCR4) is expressed on native cardiomyocytes and can modulate isolated cardiomyocyte contractility. This study examines the role of CXCR4 in cardiomyocyte response to ischaemia-reperfusion (I/R) injury. Isolated adult rat ventricular cardiomyocytes were subjected to hypoxia/reoxygenation (H/R) to simulate I/R injury. In response to H/R injury, the decrease in CXCR4 expression was associated with dysfunctional energy metabolism indicated by an increased adenosine diphosphate/adenosine triphosphate (ADP/ATP) ratio. CXCR4-overexpressing cardiomyocytes were used to determine whether such overexpression (OE) can prevent bio-energetic disruption-associated cell death. CXCR4 OE was performed with adenoviral infection with CXCR4 encoding-gene or non-translated nucleotide sequence (Control). The increased CXCR4 expression was observed in cardiomyocytes post CXCR4-adenovirus transduction and this OE significantly reduced the cardiomyocyte contractility under basal conditions. Although the same extent of H/R-provoked cytosolic calcium overload was measured, the hydrogen peroxide-induced decay of mitochondrial membrane potential was suppressed in CXCR4 OE group compared with control group, and the mitochondrial swelling was significantly attenuated in CXCR4 group, implicating that CXCR4 OE prevents permeability transition pore opening exposure to overload calcium. Interestingly, this CXCR4-induced mitochondrial protective effect is associated with the enhanced signal transducer and activator of transcription 3 (expression in mitochondria. Consequently, in the presence of H/R, mitochondrial dysfunction was mitigated and cardiomyocyte death was decreased to 65% in the CXCR4 OE group as compared with the control group. I/R injury leads to the reduction in CXCR4 in cardiomyocytes associated with the dysfunctional energy metabolism, and CXCR4 OE can alleviate mitochondrial dysfunction to improve cardiomyocyte survival.

## Introduction

Coronary heart disease is the leading cause of death in the world 1. Although the restoration of blood flow in narrowed or blocked coronary arteries can be achieved using fibrinolytics, thrombolytics, or angioplasty procedures, reperfusion after total coronary occlusion can lead to sustained myocardial injuries such as arrhythmia, apoptosis and necrosis 1,2. Actually, reperfusion initiates a cascade of events within the first few minutes after restoration of flow, and this complex procedure is contributed by multiple signalling pathways and molecular players 3. Therefore, it is important to delineate underlying cellular and molecular mechanisms contributing to heart muscle cell (cardiomyocyte) injury following ischaemia-reperfusion (I/R).

Chemokine (C-X-C motif) receptor 4 (CXCR4) is a G protein-coupled chemokine receptor expressed in a myriad of cell types with demonstrated ligand specificity for endogenous stromal derived factor-1α (SDF-1α) 4. Activation of SDF-1α/CXCR4 signalling axis plays a critical role in many cellular events, including the development of tumours and metastasis 4, and the regulation of inflammation by endowing lymphocytes with potent chemotactic activity 5. Recent studies indicate that CXCR4 can also regulate adult cardiomyocyte contractility by alteration of calcium channel activity on the cell membrane accompanied by an interaction with β_2_-adrenergic receptors 6,7. Clinical investigation has revealed up-regulation of both SDF-1α and CXCR4 in cardiac tissue from patients with end-stage heart failure 8, implying the important role of this signal axis in the development of human heart failure. Although some *in vivo* experimental evidence have been provided that SDF-1α/CXCR4 up-regulation may exacerbate the cardiac dysfunction through recruitment of inflammatory cells, promoting tumour necrosis factor-alpha secretion, and activation of cell death/apoptotic pathways 9, the CXCR4 activation-induced cardiac protective effects have been observed *ex vivo* in acute global cardiac I/R 10. In addition, CXCR4 gene transfer can prevent pressure overload induced heart failure in murine model 11, implying that myocardial CXCR4 up-regulation may serve as a protective molecular mechanism in response to various myocardial stress conditions.

Mitochondrial dysfunction plays a key role in the pathogenesis of I/R injury 12, and it is important to elucidate the cellular and molecular mechanisms involved in the mitochondrial protection mechanism upon detrimental stimuli. Fortunately, major progress has been made in deciphering mechanisms to protect mitochondrial function, and mitochondrial-targeted molecules have been identified to protect against I/R injury. In this study, the time-dependent reduction in CXCR4 was observed in isolated rat cardiomyocytes after exposure to I/R injury, associated with disorder in energetic metabolism. CXCR4 was thereby overexpressed in rat cardiomyocytes by adenovirus to investigate whether CXCR4 overexpression (OE) can alleviate I/R-induced cardiomyocyte injury through regulating mitochondrial function.

## Materials and methods

### Rat ventricular cardiomyocyte isolation and culture

The animals were handled in accordance with the Guide for the Care and Use of Laboratory Animals published by the US National Institutes of Health (NIH Publication No. 85-23, revised 1996) and the National Research Council Guide for the Care and Use of Laboratory Animals: 8th Edition published by The National Academies Press, 2011, Washington, DC. All animal experimental protocols were approved by the Institutional Animal Care and Use Committee of the University of Cincinnati (Protocol No. 06-03-03-01). Sprague–Dawley rats weighing 250–300 g were anaesthetized by a combined intraperitoneal injection of ketamine (90 mg/kg bw) and xylazine (15 mg/kg bw). The adequacy of anaesthesia was evaluated by monitoring hindlimb reflexes. When unconscious state was induced, rat hearts were excised from the thoracic cavity and the ventricular cardiomyocytes were isolated and cultured as previously described 6. Following previous protocols 6,13, CXCR4-containing adenovirus was constructed and infected in to rat cardiomyocytes, and the cardiomyocytes were exposed to SDF-1α (125 ng/ml) before experiment.

### Cardiomyocyte contractility measurements

Cardiomyocytes that adhered to the coverslips were equilibrated in KHB containing 1 mM Ca^2+^ for 20 min. at 37°C, as previously described 6. The cardiomyocyte suspension was then placed in a Plexiglas chamber, which was positioned on the stage of an inverted epifluorescence microscope (Diaphot 200; Nikon, Tokyo, Japan). Cardiomyocyte contraction was field-stimulated by a Grass S5 stimulator (0.5 Hz, square waves; Grass Technologies, An Astro-Med, Inc., West Warwick, RI, USA), and contractions were videotaped and digitized on a computer. A video edge motion detector (Crescent Electronics, Windsor, ON, Canada) was used to measure cardiomyocyte length and cell shortening, from which the per cent fractional shortening (% FS) and maximal rates of contraction and relaxation (±dl/dt) were calculated 13. All data were analyszed using software from Felix 1.1 software (Photon Technology International, Birmingham, NJ, USA) and IonWizard (IonOptix Corp., Milton, MA, USA).

### Cytosolic Ca^2+^ measurements following hypoxia-reoxygenation

Cytosolic Ca^2+^ was measured as previously described with modifications 14. Isolated cells were exposed to hypoxic conditions of 1% O_2_ and 20% CO_2_ while the culture media was changed with a hypoxic buffer for 3 hrs, followed by 4-hr reoxygenation. After hypoxia/reoxygenation (HR) treatment, myocytes were loaded with 5 μM asante calcium red (ACR; cat. no. 3010; TEFLABS Inc, Austin, TX, USA) for 30 min. at 37°C, washed three times, and harvested as a cell suspension for cytosolic Ca^2+^ measurements using flow cytometry. Fluorescence signals from ACR loaded cells excited at 540 nm were collected at an emission wavelength of >650 nm. The total fluorescence intensity was analysed from approximately 10,000 viable cardiomyocytes per sample. FlowJo software (Tree Star Inc., Ashland, OR, USA) was used to generate histograms of cell distribution according to fluorescence intensity, and to calculate the arithmetic mean of ACR intensity as an indication of cytosolic Ca^2+^ concentration.

### Assessment of cardiomyocyte apoptosis

Cardiomyocytes were prepared as previously described with modifications 15. Briefly, apoptosis was induced by simulating I/R in cardiomyocytes first incubated in ischaemia buffer at 20% CO_2_, 1% O_2_ and 37°C for 3 hrs. The solution was then switched to 1.13 mmol/l Ca-Tyrode’s solution and cell suspensions were kept at 5% CO_2_, 20% O_2_ at 37°C. For CXCR4 expression level assessment, the reoxygenation time of cardiomyocyte samples was stopped at 1, 4, 7 and 9 hrs. For cell death analysis, cardiomyocytes were harvested at 4-hr post reoxygenation treatment. Briefly, cells were gently washed once and stained with Annexin V-conjugated dye (eBioscience Inc., San Diego, CA, USA) for 25 min. Stained cardiomyocytes were then washed gently once and re-suspended in solution containing eFluor-780 (eBioscience Inc., San Diego, CA, USA). Fluorescence signals from PE-Cy7 and eFluor-780 excited at 488 and 633 nm were collected at emission wavelengths of 767 and 780 nm, respectively, in >10,000 cardiomyocytes per sample group. FlowJo software (Tree Star Inc.) was used to generate a cell distribution diagram according to fluorescence intensity, and to calculate the per cent of Annexin V positive apoptotic cells in the total population. In addition, DNA fragmentation was performed in cardiac lysates using a Cell Death Detection ELISA plus kit (cat. no. 11774425001; Roche, Indianapolis, IN, USA), which quantified the cytoplasmic histone-associated DNA fragments 15.

### ADP and ATP measurement

Cardiomyocyte intracellular ADP and ATP levels were measured using an ADP colorimetric assay kit (cat. no. K355-100; BioVision, Milpitas, CA, USA) and an ATP colorimetric assay kit (cat. no. K354-100; BioVision, Milpitas, CA, USA), according to the manufacturer’s instructions. The optical density was recorded by spectrophotometer at 570 nm and the values were expressed as the ADP/ATP ratio.

### Mitochondrial membrane potential (Δψm)

Stromal derived factor-1α (125 ng/ml)-treated cardiomyocytes were loaded with tetramethylrhodamine, ethyl ester, perchlorate (10 nM) (TMRE, cat. no. T669; Invitrogen, Grand Island, NY, USA) at 37°C for 30 min. After exposure to hydrogen-dioxide, the loaded cells were excited at 568 nm and the magnitude of the emitted fluorescence was measured at 630 nm, the TMRE intensity was recorded by flow-cytometer according to the cell number distribution. Finally, the FlowJo software (Tree Star Inc.) was used to calculate the mean integral fluorescence density to indicate Δψm.

### Measurement of mitochondrial swelling

Cardiomyocytes were gently homogenized in 1.5 ml buffer (128 mmol/l Sucrose 10 mmol/l Tris, 1.0 mmol/l EDTA with protease inhibitor, pH = 7.4) and centrifuged at 1000 × g for 5 min. The supernatant was centrifuged at 13,000 × g for 20 min. at 4°C and the pellet was re-suspended with 1 ml washing buffer (250 mmol/l Sucrose 10 mmol/l Tris, pH = 7.4) to obtain the mitochondria. The mitochondria pellet was then re-suspended in a swelling buffer (120 mmol/l KCl, 10 mmol/l Tris, 5 mmol/l KH_2_PO_4_, pH = 7.4) and incubated on ice for 2 hrs. Mitochondria (200 μl, 250 μg/μl) was re-suspended in 96-well plates and incubated at room temperature for 5 min. The absorbance at 520 nm was recorded in the absence or presence of Ca^2+^ (250 μmol/l) with a spectrophotometer. Cyclosporine A (1 μM) was added into Ca^2+^-loaded mitochondrial to be treated as the control of mitochondrial permeability transition pore.

### Western blot

Western blot analysis was performed as previously described 16. Membranes were blocked with 5% milk and then probed with specific antibodies against CXCR4 (cat. no. AB1847; Millipore, Temecula, CA, USA), signal transducer and activator of transcription 3 (STAT3; cat. no. 4904; Cell Signaling Technology, Danvers, MA, USA), COX IV (cat. no. 62164; Abcam, Cambridge, MA, USA), SERCA2a (Affinity Bioreagents) glyceraldehyde-3-phosphate dehydrogenase (GAPDH; Sigma-Aldrich, St. Louis. MO, USA), total phospholamban (PLN) and total RyR (Thermo Scientific, Rockford., IL, USA), phospho-ser16-PLN, phospho-ser2809-RyR (Badrilla, Leeds, West Yorkshire, UK), The ECL system (GE Healthcare Biosciences, Pittsburgh, PA, USA) was used for detection.

### Statistical analysis

Data were expressed as mean ± SEM. Multiple comparisons among three or more groups were performed with one-way anova, and the Bonferroni exact test was applied for *post hoc* analyses (SPSS 13.0., IBM Co., Armonk, NY, USA). A value of *P* < 0.05 was considered statistically significant.

## Results

### Cardiomyocytes CXCR4 expression decreased in response to H/R injury

**Figure 1:**
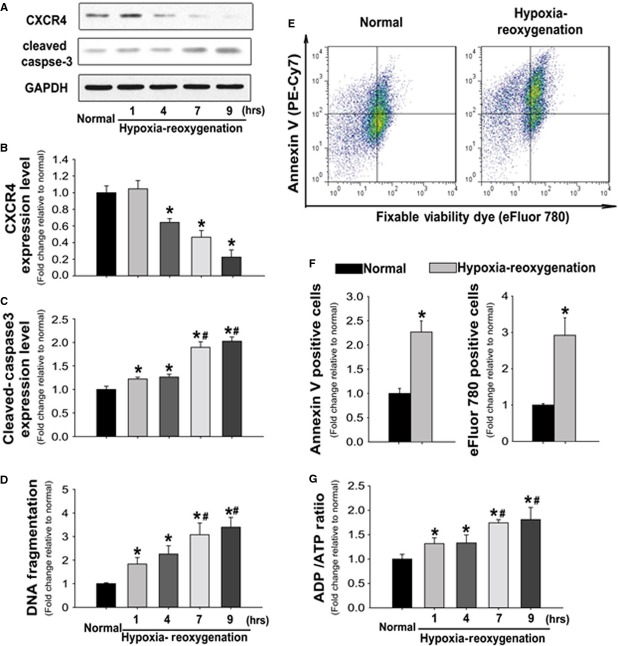
Hypoxia-reoxygenation-induced apoptosis is associated with the decreased CXCR4 expression in rat cardiomyocytes. (A) Representative immunoblots illustrating CXCR4, cleaved-caspase3 and GAPDH in response to the hypoxia/reoxygenation (H/R). (B and C) Quantitative analysis of CXCR4 (B) and cleaved-Caspase3 (C) expression levels relative to normal condition after normalization to GAPDH levels. (D) DNA fragmentation (mono- and oligo-nucleosomes contents) in isolated rat myocytes was detected by ELISA in presence and absence of H/R injury. (E) Representative flow cytometric pseudo-colour density plots illustrating the distribution of apoptotic (PE-Cy7 positive) and necrotic (eFluor-780 positive) cells. (F) Quantitative analysis of annexin V or eFluor-780 positive cells in either the absence or presence of HR injury. (G) ADP and ATP concentrations were assessed by colorimetric assay, and the ratios of ADP to ATP were used to determine the energetic metabolism of cardiomyocytes either in the absence or presence of H/R. *n* represents six independent experiments; *P < 0.05 *versus* normal group; ^#^*P* < 0.05 *versus* H/R 1 and 4 hrs.

Isolated rat cardiomyocytes were subjected to H/R condition to investigate the alteration of CXCR4 expression level in response to I/R injury. After exposure to 3 hrs of hypoxia and 1 hr of re-oxygenation, CXCR4 expression level was not changed significantly. However, the expression level was decreased by 36%, 55% and 77% after re-oxygenation for 4, 7 and 9 hrs, respectively, when compared to cardiomyocytes maintained under normal control conditions (Fig.1A and B). Immunoblot analysis indicated that the appearance of the cleaved form of caspase-3 was enhanced to 1.20-, 1.23-, 1.80- and 2.0-fold in response to 1, 4, 7 and 9 hrs of re-oxygenation compared to normal status (Fig.1C). During apoptosis, activated caspase-3 can cleave nuclear DNA between nucleosomes, producing a mixture of DNA fragments. Consistent with this, the ELISA assay showed that the DNA fragmentation increased in a time-dependent pattern post H/R (Fig.1D). Indeed, both apoptosis and necrosis were increased which demonstrated by the enhanced Annexin V and eFluor-780 positive cells respectively (Fig.1E and F). Cellular bioenergetics, as is indicated by the ADP/ATP ratio, also increased in a time-dependent manner after cardiomyocyte exposure to H/R injury (Fig.1G).

### Adenoviral overexpressing CXCR4 in rat cardiomyocytes

**Figure 2:**
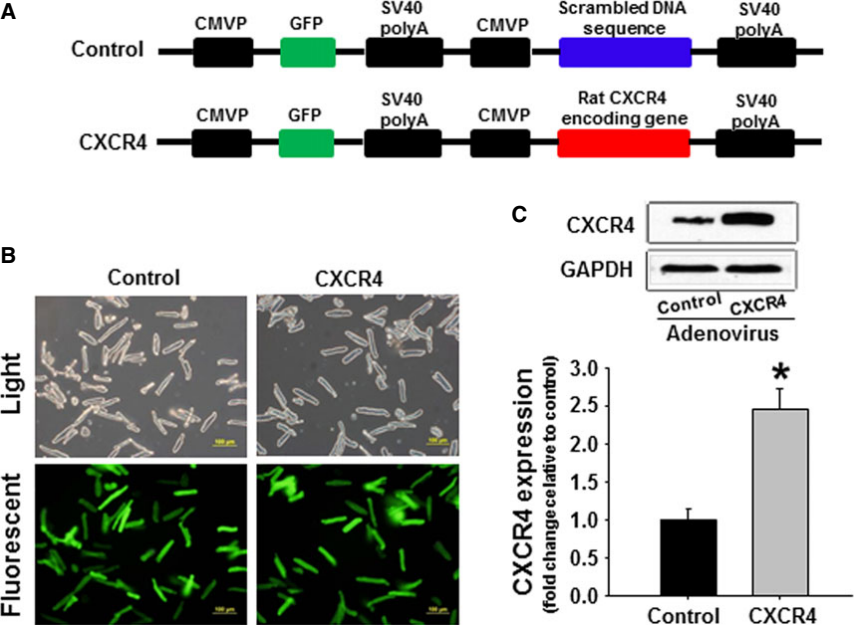
Preparation of CXCR4-overexpressing rat cardiomyocyte. (A) Schematic diagram of the recombinant adenoviral vector. The rat CXCR4-encoding gene as well as a scrambled DNA sequence were amplified with PCR and incorporated into CMV downstream in the Adeasy-1/shuttle backbone vector. (B) Cardiomyocytes appeared infected by nearly 100% after 48-hr adenoviral gene transfer, as indicated by the appearance of GFP fluorescence. No morphological changes were detected in control- and CXCR4-adenovirus-infected cells. (C) Representative Western blots and quantitative data analysis illustrating the expression level of CXCR4 in rat cardiomyocytes after CXCR4-adenoviral gene transfer. (*n* represents six independent experiments; *P < 0.05).

To investigate the potential effects of CXCR4 in cardiomyocytes, we generated an adenovirus containing the rat CXCR4 encoding gene and an adenovirus containing the non-translated nucleotide sequence as control (Fig.2A). Both viruses also contained the GFP gene. Nearly 100% of ventricular cardiomyocytes appeared infected after 48 hrs, as indicated by green fluorescence (Fig.2B). Western blots confirmed that the expression level of CXCR4 was increased by 2.5-fold in CXCR4-adenoviral infecting group when compared to control group (Fig.2C), and CXCR4-overexpressing cardiomyocytes elicited the decreased contractile function (Fig. S1) and the compromised calcium handling (Fig. S2) upon the stimulation of SDF-1α.

### CXCR4 overexpression protected against overload calcium- and oxidative stress-induced mitochondrial dysfunction

**Figure 3:**
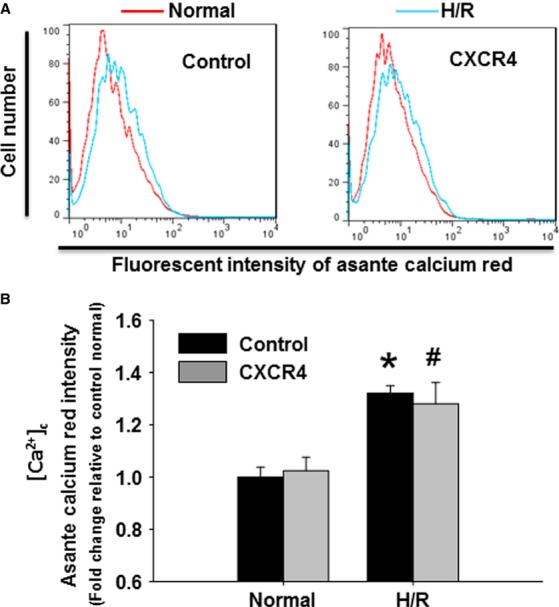
Cytosolic calcium overload in response to hypoxia/reoxygenation. (A) Representative flow cytometry histogram illustrating the distribution of asante calcium red (ACR) positive cardiomyocytes in the presence or absence of H/R. (B) Quantitative analysis of cytosolic Ca^2+^ concentration ([Ca^2+^]_c_) based on ACR fluorescence intensities, which are expressed as fold change relative to control group under normal condition. *n* represents six independent experiments; *P < 0.05 *versus* control group under normal condition; ^#^*P* < 0.05 *versus* CXCR4 group under normal condition.

Calcium overload is a cellular consequence of I/R injury resulting from a disorder in cell membrane calcium ion transporting mechanisms. This calcium overload can subsequently damage functions of cellular organelles and initiate cell death. Under basal conditions, there was no difference in cytosolic calcium concentration between control and CXCR4 group (Fig.3A and B). Calcium concentration was increased 1.35-fold in the control group compared to normal conditions in response to H/R. Although calcium overload was also induced in CXCR4 group, there was no statistical difference between control and CXCR4 overexpressing group under H/R conditions.

**Figure 4:**
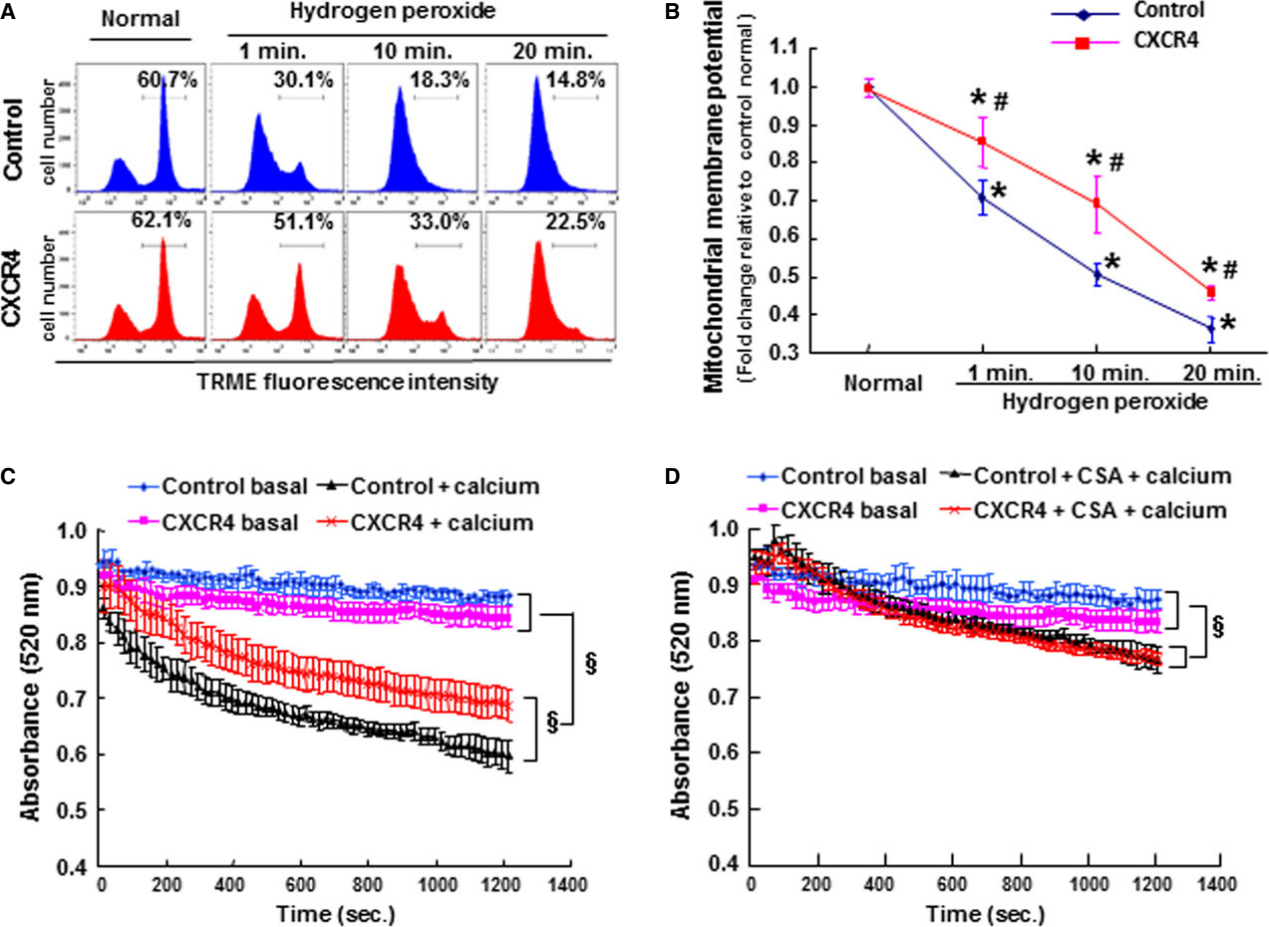
CXCR4 overexpression attenuated mitochondrial dysfunction in response to stress stimuli. (A) Representative histogram illustrating the distribution of TMRE-loaded cardiomyocytes according to the fluorescence intensity. (B) Quantitative analysis of the mean of TMRE intensity to illustrate mitochondrial membrane potential, which are expressed as fold change relative to control group under normal condition. (C) Absorbance at 520 nm was recorded to illustrate mitochondrial swelling either in the presence or absence of Ca^2+^ (250 nmol/l). (D) Absorbance at 520 nm was monitored to illustrate mitochondria swelling after Ca^2+^ stimulation in the presence of cyclosporine A (CSA; 1 μM) *n* represents six independent experiments; *P < 0.05 *versus* control group under normal condition; ^#^*P* < 0.05 *versus* control group at the same time-point; ^§^*P* < 0.05.

Elevated cytosolic calcium and oxidative stress will induce excessive mitochondrial Ca^2+^ entry, which can initiate mitochondrial permeability transition pore (mPTP) associating with dissipating the mitochondrial inner membrane potential (Δψm). Under basal conditions, Δψm was similar in control and CXCR4 overexpressing cardiomyocytes, as seen by the strong TMRE fluorescent intensity distribution (Fig.4A). In the presence of oxidative stress induced by hydrogen peroxide, the TMRE fluorescence intensity dissipated in a time-dependent manner (Fig.4A). Quantitative analysis of the flow cytometer data indicated that Δψm decreased by 30%, 50% and 64% at 1, 10 and 20 min. after hydrogen peroxide treatment, when compared to the basal condition. The reduction percentage of Δψm at the same points in CXCR4-transduced cardiomyocytes was 15%, 31% and 55% (Fig.4B).

The disruption of mitochondrial membrane potential is accompanied by swelling as a consequence of opening of the mPTP, and this organelle disruption can be detected through consecutively recorded light absorbance at 520 nm wavelength. In the absence of calcium, the absorbance of isolated mitochondria was ∼0.9 in both the control group and CXCR4-transduced cardiomyocytes (Fig.4C). After 20 min. exposure to 250 μM Ca^2+^ the absorbance of mitochondria in control group decreased from 0.9 to 0.6, while this parameter in CXCR4 over-expressing group decreased less, from 0.9 to 0.7 (Fig.4C). Interestingly, the Ca^2+^-induced mitochondrial swelling was mitigated to the same level between control group and CXCR4 group, after blockade of mPTP by 1 μM cyclosporine A (Fig.4D).

### Mitochondrial STAT3 expression

**Figure 5:**
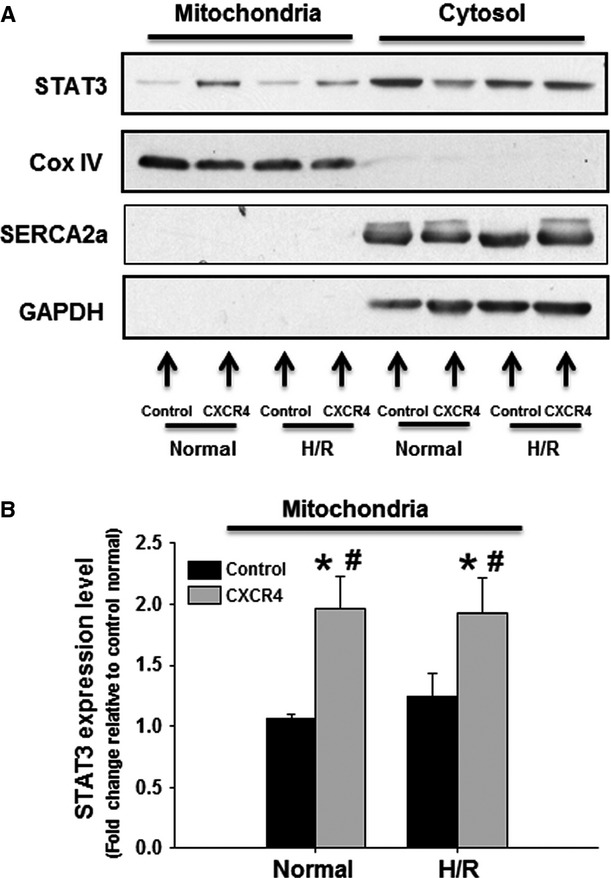
Mitochondrial STAT3 increased in CXCR4-overexpressing cardiomyocytes. (A) Representative Western blots illustrating the expression of STAT3, CoxIV, SERCA2a and GAPDH in mitochondrial- and cytosolic fraction. (B) Quantitative analysis of mitochondrial STAT3 expression level either in the absence or presence of H/R. *n* represents six independent experiments; *P < 0.05 *versus* control under normal condition, ^#^*P* < 0.05 *versus* control under H/R condition.

Cardiomyocyte mitochondria were isolated to examine further the possible mechanism underlying these CXCR4 OE-induced mitochondrial protective effects. The purification of isolated mitochondria was determined by the appearance of Cox IV using Western blot analysis, and this was confirmed without the detection of SERCA2a (SR marker) and GAPDH (cytosolic contaminant marker) in mitochondrial portion (Fig.5A). Both under basal and H/R conditions, the mitochondrial STAT3 expression level was enhanced to a greater extent in CXCR4 overexpressing cardiomyocytes than in the control group (Fig.5A and B).

### CXCR4 overexpression reduced hypoxia-induced cardiomyocyte death

**Figure 6:**
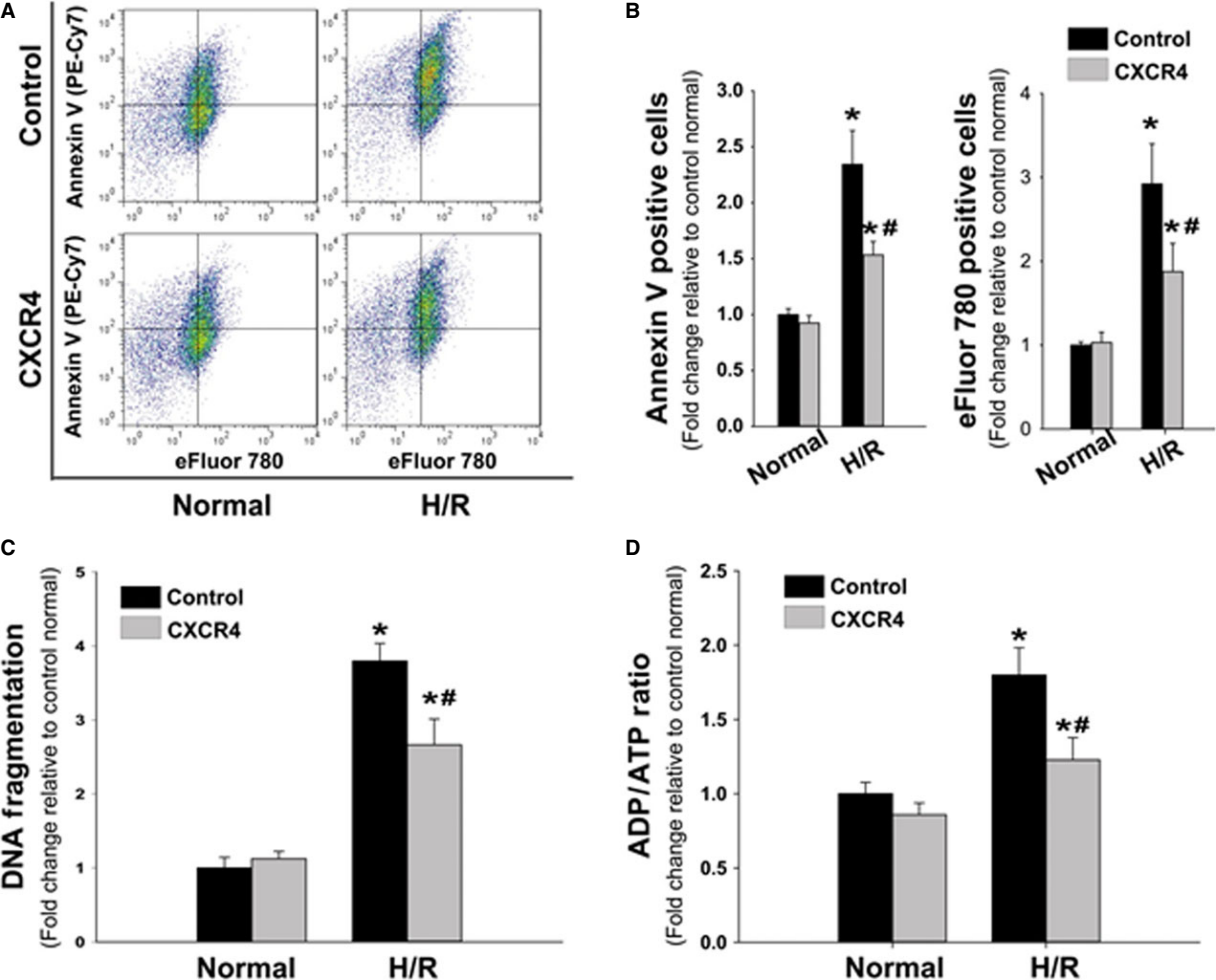
Overexpression of CXCR4 reduced the HR-induced the death of cardiomyocyte. (A) Representative flow-cytometric pseudo-colour density plots illustrating the distribution of apoptotic and necrotic cardiomyocytes at the basal or HR condition, after either infections of Control- or CXCR4-adenoviral infection. (B) Quantitative analysis of annexin V and eFluor-780 positive cells, (C) DNA fragmentation and (D) the ratios of ADP to ATP under normal and HR condition, in response to Control- or CXCR4- adenoviral infection. *n* represents six independent experiments *P < 0.05 *versus* control under normal condition, ^#^*P* < 0.05 *versus* control under H/R condition.

Transfected myocytes were subjected to H/R to assess any potential alterations elicited by the CXCR4 OE based on an earlier report that activation of SDF-1α/CXCR4 axis can mediate acute cardioprotection in response to global I/R 10. Results showed 2.3-fold augmentation in Annexin V positive cardiomyocytes, as well as 2.9-fold augmentation in eFluor-780 positive cardiomyocytes, in control Ad.GFP-infected cells following H/R. However, the fold changes of Annexin V (apoptotic) and eFluor-780 (necrotic) cells in the CXCR4 group were significantly lower than control group after H/R (Fig.6A and B). These findings were further supported by results obtained with the DNA fragmentation assay. There was 3.7-fold increase in the extent of DNA fragmentation in the control group, while this increase was suppressed in CXCR4 overexpressing group post H/R injury (Fig.6C). Finally, an increase in the ADP/ATP ratio indicated the H/R-induced energy metabolism disorder was significantly reduced in CXCR4 group (Fig.6D).

### Inhibition of mitochondrial STAT3 compromised the CXCR4-induced protective effects in cardiomyocytes response to H/R injury

**Figure 7:**
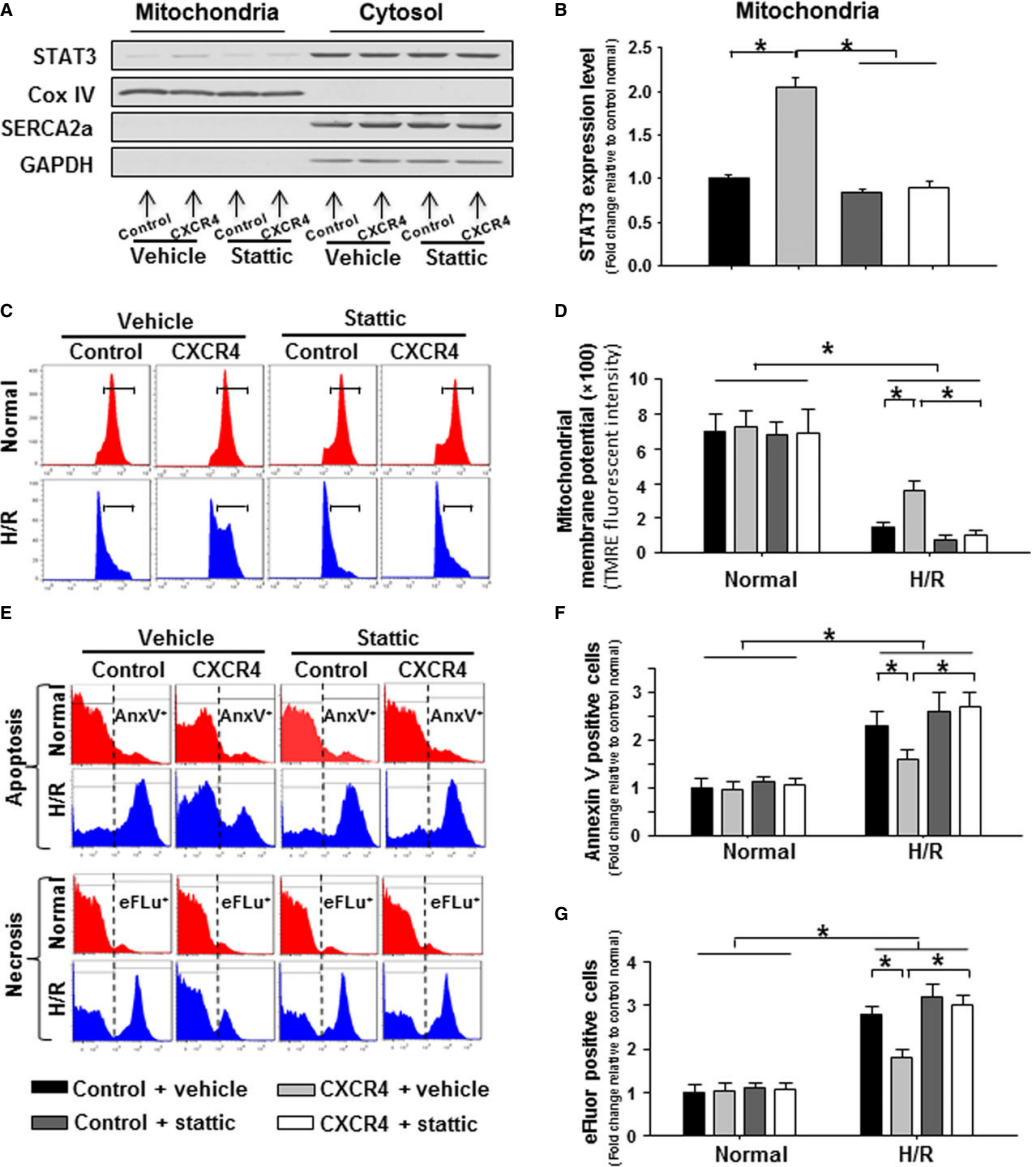
The CXCR4 overexpression-induced anti-death effects were abrogated in cardiomyocytes response to mitochondrial STAT3 inhibition. (A) Western blots illustrating the expression of STAT3, CoxIV, SERCA2a and GAPDH in mitochondrial- and cytosolic fraction in the presence of stattic (20 μM) or solvent vehicle. (B) Quantitative analysis of mitochondrial STAT3 expression level. (C) Representative flow-cytometric histogram illustrating the distribution of TMRE-loaded cardiomyocytes according to the fluorescence intensity. (D) Quantitative analysis of the mean of TMRE intensity to illustrate mitochondrial membrane potential. (E) Representative flow-cytometric histogram illustrating the distribution of AnnexinV positive (AnxV^+^) and eFluor positive (eFLu^+^) cardiomyocytes at the basal or HR condition after exposure to stattic. (F and G) Quantitative analysis of apoptotic cells (F) and necrotic cells (G). *n* represents six independent experiments; *P < 0.05.

Pharmacological inhibition of STAT3 by Stattic was used to investigate role of the enhanced mitochondrial STAT3 in CXCR4-induced protective mechanism in cardiomyocytes. As is shown in Figure7A and B, the increased mitochondrial STAT3 expression in CXCR4-overexpressing cardiomyocytes was reduced to the same level as control group in the presence of Stattic (20 μM), and this dose cannot compromise mitochondrial function (Fig.7C and D) and cell survival (Fig.7E–G) under normal condition. In the presence of H/R injury, the collapse of mitochondrial membrane potential was slow down in CXCR4-overexpressing rat cardiomyocytes, but this organelle defending effect was dismissed after exposure to Stattic (Fig.7C and D). Correspondingly, the enhanced pro-survival capacity was abrogated in CXCR4-overexpressing cardiomyocyte post mitochondrial STAT3 inhibition by Stattic, since there was no difference in apoptotic (Annexin V positive) and necrotic (eFluor positive) cell distribution between control and CXCR4 group post H/R injury (Fig.7E–G).

## Discussion

Our findings demonstrated that the simulated I/R-induced reduction in CXCR4 is associated with a disorder in energy metabolism, and that adenoviral OE of CXCR4 can protect isolated rat cardiomyocytes from H/R-induced cell death by preserving mitochondrial function. To our knowledge, this is the first study showing that the reduced-CXCR4 participates in the cardiomyocyte intracellular injury mechanism during I/R.

Cardiac I/R is a multifactorial process involving not only intracellular injury processes but also injurious inflammatory responses 2,17. CXCR4, one of chemokine receptors, is activated selectively by the endogenous ligand SDF-1α, and upon activation can regulate the migration of many different cell types, most notably immune cells 4. Such cell migration conveys a double-edged effect on the recovery of heart function. Specifically, although beneficial effects can result from neovascularization after heart tissue-specific or hematopoietic progenitor cells are redirected to the damaged site 18–20, detrimental outcome can also appear because of excessive infiltration of inflammatory cells and cytokines 9.

It is reported that SDF-1α/CXCR4 are also expressed on adult cardiomyocytes and directly regulate cardiomyocyte contractile function. Treatment with exogenous SDF-1α can blunt positive inotropic responses of cardiomyocytes to physiological concentrations of calcium, and adenoviral-transduced CXCR4 constructs accentuate the negative inotropic effects in isolated adult rat cardiomyocytes 7. We provided the *in vitro* evidence that CXCR4 expression level decreased in a time-dependent manner in response to reperfusion, and this reduction was associated with a cellular energetics disorder demonstrated by an increased ADP/ATP ratio. Interestingly, when this cellular energetic deficit was rescued by CXCR4 OE, the survival of cardiomyocytes improved, suggesting that SDF-1α/CXCR4 signal cascades may participate in the regulation of the cellular energy production under stressful conditions.

Mitochondria occupy approximately one-third of cellular volume in cardiac myocytes and play a central role in energy metabolism 21. The generation of ATP occurs predominantly through oxidative phosphorylation, and this is highly dependent on the mitochondrial transmembrane potential (Δψm) in which electrons are passed along a series of protein complexes situated in the mitochondrial inner membrane to form respiratory chain 22. Indeed, a rapid Δψm reduction in isolated cardiomyocytes was observed in the hydrogen peroxide-simulated oxidative stress condition, whereas OE of CXCR4 significantly retarded this depolarization. Importantly, although attenuation of H/R-induced Ca^2+^ overload was not observed, Ca^2+^ overload-induced mitochondrial swelling was significantly suppressed in CXCR4 overexpressing cardiomyocytes, indicating that CXCR4 OE induced cardiac protective effects were partly attributable to its regulatory effects on mitochondrial function.

Signal transducer and activator of transcription 3, a major downstream target of interleukin-6 in cardiac fibroblast 23, has recently been identified in cardiomyocyte mitochondria 24,25. Indeed, nucleus-encoded STAT3 can be imported into matrix of subsarcolemmal and interfibrillar cardiomyocyte mitochondria *via* Tom20-dependent pathway 26, and the activated form of mitochondrial STAT3 can interact with complex I/II and cyclophilin D to regulate mitochondrial oxygen consumption and mPTP opening respectively 26. In an *in situ* pig model of regional myocardial I/R, mitochondrial STAT3 activation has elicited the pronounced cardioprotective effects through improving mitochondrial function 27, while pharmacological inhibition or genetic blockade of STAT3 impair mitochondrial function and abrogate ischaemic postconditioning-induced cardioprotective effects 28,29. Our data showed that STAT3 appearance in mitochondrial fraction was enhanced in both normal and H/R conditions in response to CXCR4 OE. Interestingly, pharmacological inhibition of enhanced-mitochondrial STAT3 significantly abrogated the CXCR4-induecd mitochondrial protective effects and consequently compromised the survival of CXCR4-overexpressing cardiomyocytes, suggesting that SDF-1α/CXCR4-mediated anti-cell death effects were partially a result of the activation of a mitochondrial STAT3 protective mechanism.

However, a significant limitation of present study is lack of *in vivo* evidence to confirm the role of SDF-1α/CXCR4 signal axis in maintaining mitochondrial function. Thus, it will be valuable to perform gain-and-loss of function experiments *in vivo* and obtain more detailed information on how this membrane receptor regulates mitochondrial function. The hearts from CXCR4 transgenic and knockout mice can be subjected to I/R, which may closer mimic the corresponding condition in human disease and elucidate the underlying mechanisms.

In conclusion, CXCR4 expression level decreases in response to I/R injury. Under stress conditions, when CXCR4 is overexpressed in cardiomyocytes by adenoviral transfection, mitochondrial membrane potential is somewhat protected from collapse and the opening of the mitochondrial permeability pore is decreased, which together relieve the disordered cellular energy metabolism *via* activating STAT3 signalling pathway. In the light of recent finding on beneficial effects elicited by CXCR4 gene transfer in pressure overload-induced heart failure 11, as well as ongoing clinical trial of adeno-associated virus-mediated gene therapy for heart failure 30, it is interesting to propose that CXCR4 gene therapy may serve as a potential preventive and therapeutic approach for I/R injury during revascularization treatment or cardiac transplantation.

## Supporting Information

**Figure S1** Over-expression of CXCR4 suppresses rat cardiomyocyte contractility. **Figure S2** Alterations of calcium handling in rat cardiomyocytes in response to CXCR4 over-expression.

## References

[b1] Yellon DM, Hausenloy DJ (2007). Myocardial reperfusion injury. N Engl J Med.

[b2] Hausenloy DJ, Yellon DM (2013). Myocardial ischemia-reperfusion injury: a neglected therapeutic target. J Clin Invest.

[b3] Ovize M, Baxter GF, Di Lisa F (2010). Postconditioning and protection from reperfusion injury: where do we stand? Position paper from the Working Group of Cellular Biology of the Heart of the European Society of Cardiology. Cardiovasc Res.

[b4] Wu B, Chien EY, Mol CD (2010). Structures of the CXCR4 chemokine GPCR with small-molecule and cyclic peptide antagonists. Science.

[b5] Duchesneau P, Gallagher E, Walcheck B (2007). Up-regulation of leukocyte CXCR4 expression by sulfatide: an L-selectin-dependent pathway on CD4+ T cells. Eur J Immunol.

[b6] LaRocca TJ, Schwarzkopf M, Altman P (2010). β2-Adrenergic receptor signaling in the cardiac myocyte is modulated by interactions with CXCR4. J Cardiovasc Pharmacol.

[b7] Pyo RT, Sui J, Dhume A (2006). CXCR4 modulates contractility in adult cardiac myocytes. J Mol Cell Cardiol.

[b8] Damås JK, Eiken HG, Oie E (2000). Myocardial expression of CC- and CXC-chemokines and their receptors in human end-stage heart failure. Cardiovasc Res.

[b9] Chen J, Chemaly E, Liang L (2010). Effects of CXCR4 gene transfer on cardiac function after ischemia-reperfusion injury. Am J Pathol.

[b10] Huang C, Gu H, Zhang W (2011). SDF-1/CXCR4 mediates acute protection of cardiac function through myocardial STAT3 signaling following global ischemia/reperfusion injury. Am J Physiol Heart Circ Physiol.

[b11] Larocca TJ, Jeong D, Kohlbrenner E (2012). CXCR4 gene transfer prevents pressure overload induced heart failure. J Mol Cell Cardiol.

[b12] Walters AM, Porter GA, Brookes PS (2012). Mitochondria as a drug target in ischemic heart disease and cardiomyopathy. Circ Res.

[b13] Zhang D, Fan GC, Zhou X (2008). Over-expression of CXCR4 on mesenchymal stem cells augments myoangiogenesis in the infarcted myocardium. J Mol Cell Cardiol.

[b14] Cai WF, Pritchard T, Florea S (2012). Ablation of junctin or triadin is associated with increased cardiac injury following ischaemia/reperfusion. Cardiovasc Res.

[b15] Lam CK, Zhao W, Cai W (2013). Novel role of HAX-1 in ischemic injury protection involvement of heat shock protein 90. Circ Res.

[b16] Chen G, Zhou X, Florea S (2010). Expression of active protein phosphatase 1 inhibitor-1 attenuates chronic beta-agonist-induced cardiac apoptosis. Basic Res Cardiol.

[b17] de Groot H, Rauen U (2007). Ischemia-reperfusion injury: processes in pathogenetic networks: a review. Transplant Proc.

[b18] Liang J, Huang W, Yu X (2012). Suicide gene reveals the myocardial neovascularization role of mesenchymal stem cells overexpressing CXCR4 (MSC(CXCR4)). PLoS ONE.

[b19] Davidson SM, Selvaraj P, He D (2013). Remote ischemic preconditioning involves signaling through the SDF-1α/CXCR4 signaling axis. Basic Res Cardiol.

[b20] Przyklenk K (2013). ‘Going out on a limb’: SDF-1α/CXCR4 signaling as a mechanism of remote ischemic preconditioning?. Basic Res Cardiol.

[b21] Kolwicz SC, Purohit S, Tian R (2013). Cardiac metabolism and its interactions with contraction, growth, and survival of cardiomyocytes. Circ Res.

[b22] Jassem W, Heaton ND (2004). The role of mitochondria in ischemia/reperfusion injury in organ transplantation. Kidney Int.

[b23] Müller J, Gorressen S, Grandoch M (2014). Interleukin-6-dependent phenotypic modulation of cardiac fibroblasts after acute myocardial infarction. Basic Res Cardiol.

[b24] Tammineni P, Anugula C, Mohammed F (2013). The import of the transcription factor STAT3 into mitochondria depends on GRIM-19, a component of the electron transport chain. J Biol Chem.

[b25] Wegrzyn J, Potla R, Chwae YJ (2009). Function of mitochondrial Stat3 in cellular respiration. Science.

[b26] Boengler K, Hilfiker-Kleiner D, Heusch G (2010). Inhibition of permeability transition pore opening by mitochondrial STAT3 and its role in myocardial ischemia/reperfusion. Basic Res Cardiol.

[b27] Heusch G, Musiolik J, Gedik N (2011). Mitochondrial STAT3 activation and cardioprotection by ischemic postconditioning in pigs with regional myocardial ischemia/reperfusion. Circ Res.

[b28] Boengler K, Ungefug E, Heusch G (2013). The STAT3 inhibitor stattic impairs cardiomyocyte mitochondrial function through increased reactive oxygen species formation. Curr Pharm Des.

[b29] Boengler K, Buechert A, Heinen Y (2008). Cardioprotection by ischemic postconditioning is lost in aged and STAT3-deficient mice. Circ Res.

[b30] Hajjar RJ, Zsebo K, Deckelbaum L (2008). Design of a phase 1/2 trial of intracoronary administration of AAV1/SERCA2a in patients with heart failure. J Card Fail.

