# Supplementary material for: CXCR4 attenuates cardiomyocytes mitochondrial dysfunction to resist ischaemia-reperfusion injury
Source: J Cell Mol Med. 2015 Mar 30;19(8):1825–35. doi: 10.1111/jcmm.12554 (PMC4549033; doi:10.1111/jcmm.12554)
Supplement: Supplementary file 1 [file jcmm0019-1825-sd1.doc]

**Supplemental Data**

**Over-expression of CXCR4 suppresses rat cardiomyocyte contractility**

After exposure to SDF-1α, cardiomyocytes were subjected to electrical field stimulation and the contractility parameters were recorded. Under basal conditions (**Supplemental Figure 1A**), cardiomyocyte fractional shortening (FS %) (**Supplemental Figure 1B**), the rates of cell contraction (+dL/dt) (**Supplemental Figure 1C**) and relaxation (-dL/dt) (**Supplemental Figure 1D**) were reduced by 36%, 33% and 44%, respectively, in the presence of CXCR4 over-expression compared to the control group.

**Alterations of calcium handling in rat cardiomyocytes in response to CXCR4 over-expression**

Calcium transient is defined as a rise and subsequent decay of cytosolic calcium upon a membrane depolarizing electrical stimulus, and both the contractile force and frequency of cardiomyocytes depend on the calcium kinetics. In accordance with above contractile function results, the calcium transient amplitude was decreased by 38% **(Supplemental Figure 2A and 2B)**, while the time constant (Tau) of the calcium transient decay was prolonged **(Supplemental Figure 2A and 2B)** in CXCR4-overexpresssed cardiomyocytes when compared with control group. Ryanodine receptor (RyR) and phospholamban (PLN) are key Ca^2+^ cycling (release and re-uptake) proteins in the sarcoplasmic reticulum (SR) of cardiomyocytes, which constitute a critical regulatory mechanism governing SR calcium release and re-uptake. Phosphorylation of PLN and RyR was decreased to 45% and 65% respectively, in CXCR4-transduced cardiomyocytes compared to the control group **(Supplemental Figure 3D and 3E).**


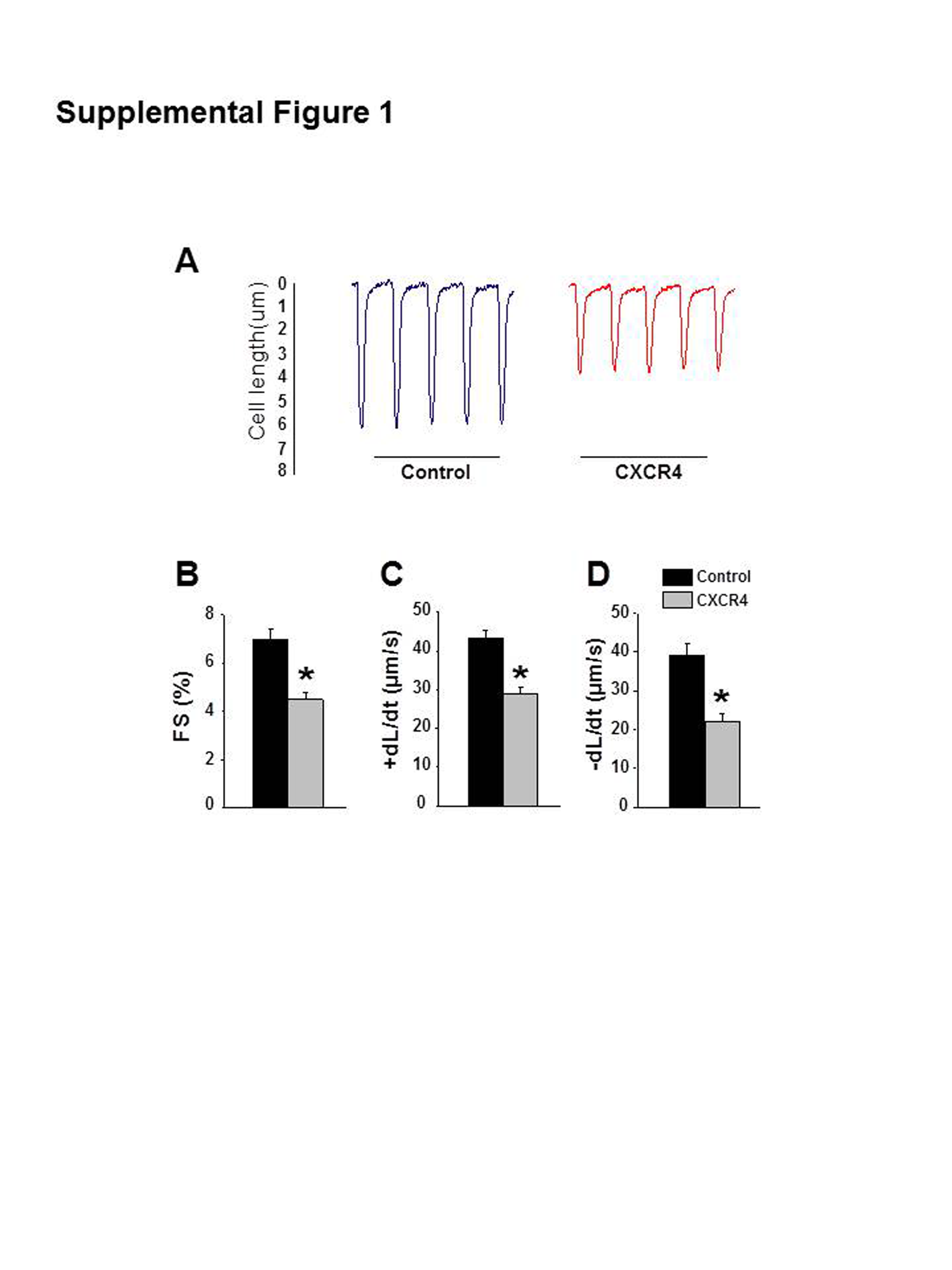


**Supplemental Figure 1. CXCR4 overexpression of suppresses the contractile function of rat cardiomyocyte. (A)** Isolated rat cardiomyocytes were suspended in 1.0mmol/L Ca^2+^ tyrode solution and field-stimulated at 0.5Hz. Representative cell-shortening traces of cardiomyocytes infected with either adenoviral-Control or adenoviral-CXCR4. **(B)** Fractional shortening (FS%), **(C)** Rates of shortening (+dL/dt) and **(D)** rates of re-lengthening (-dL/dt) were decreased when CXCR4 was over-expressed. *n*=80~90 cells from 6 hearts for each group; *P<0.05 vs. Control group

**
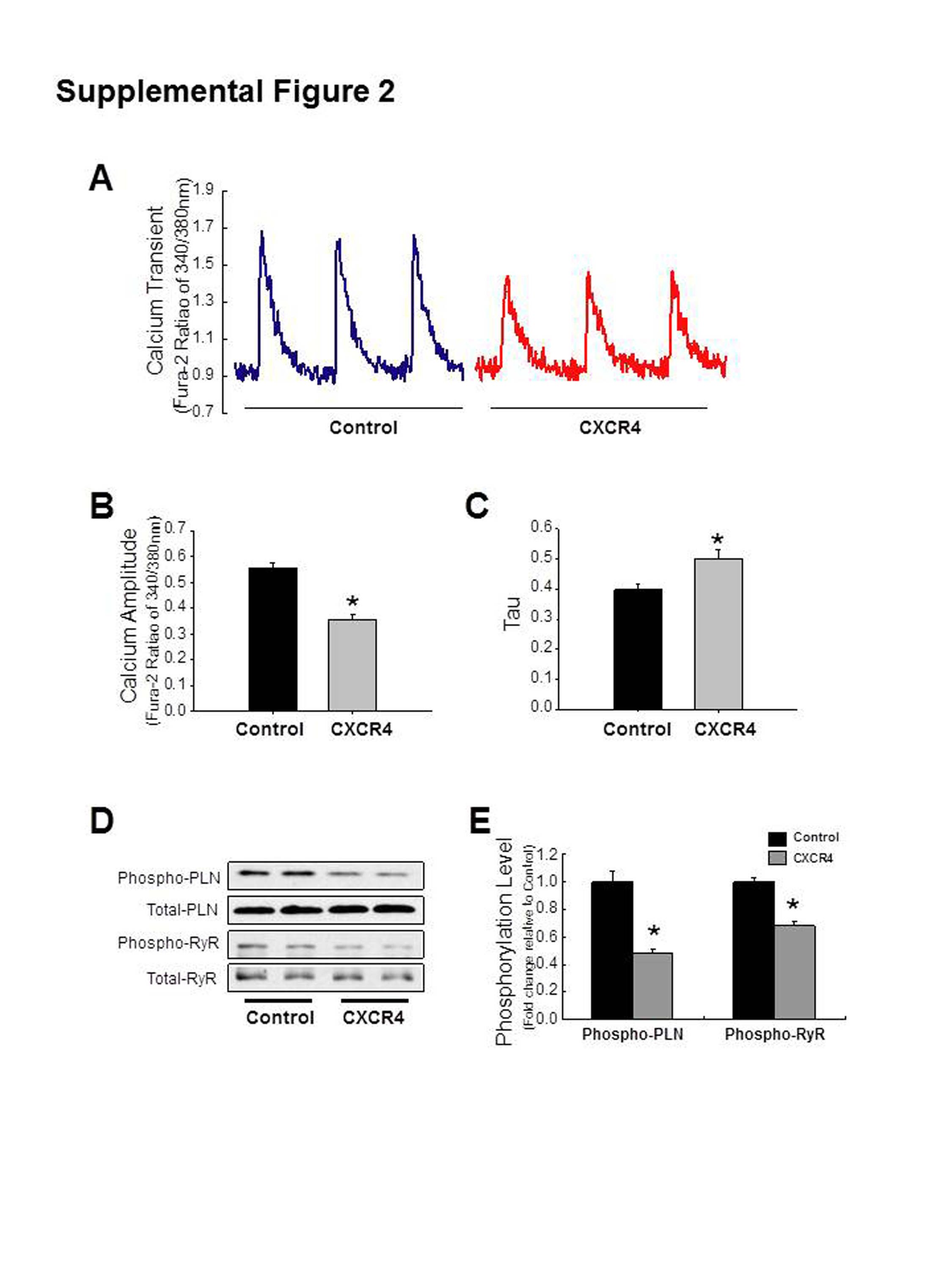
**

**Supplemental Figure 2. Effects of CXCR4 on rat cardiomyocyte calcium transients**

**(A)** Representative tracings of Ca^2+^ transients at basal conditions in the presence of either Control or CXCR4 adenovirus. **(B)** Ca^2+^ transient peak and **(C)** the time constant of the calcium transient decay (Tau). **(D)** Representative immunoblots illustrate the phosphorylation of PLN and RyR in response to the CXCR4-adenoviral infection. **(E)** Quantitative analysis of the phosphorylation levels of PLN and RyR, normalized to their total protein levels. *n*=60~80 cells from 5~6 hearts for each group; *P<0.05 vs. Control group.
